# Beliefs and Behaviors Related to Physical Activity in Black Girls With Asthma

**DOI:** 10.1002/ppul.71497

**Published:** 2026-02-05

**Authors:** Nora Spadoni, Aero Cavalier, Ellen Davis, Deborah Salvo, Shelby Langer, Sharmilee M. Nyenhuis, Anna Volerman

**Affiliations:** ^1^ Pritzker School of Medicine The University of Chicago Chicago Illinois USA; ^2^ Department of Pediatrics, Section of Allergy, Immunology, and Pediatric Pulmonology The University of Chicago Chicago Illinois USA; ^3^ Department of Kinesiology and Health Education, College of Education The University of Texas at Austin Austin Texas USA; ^4^ Edson College of Nursing and Health Innovation Arizona State University Phoenix Arizona USA; ^5^ Department of Medicine, Section of General Internal Medicine The University of Chicago Chicago Illinois USA; ^6^ Department of Pediatrics, Section of Academic Pediatrics The University of Chicago Chicago Illinois USA

**Keywords:** asthma, caregiver support, health disparities, physical activity

## Abstract

**Introduction and Objective:**

Physical activity (PA) is associated with improved asthma outcomes. Black girls face higher rates of asthma morbidity and are less likely to meet recommended PA than their White and Black male peers. To address these health disparities, it is essential to understand beliefs and behaviors related to PA among Black girls with asthma.

**Methods:**

For this qualitative study, Black girls with asthma and their mothers or female caregivers were recruited through flyers and direct outreach to patients at one academic medical center. Semi‐structured interviews focused on knowledge of PA recommendations, perceived risks and benefits of PA, barriers and facilitators to PA, and maternal influences on PA. Transcripts were coded iteratively through deductive thematic analysis.

**Findings:**

Twenty girls (age: mean = 9.9 years, SD = 1.33, range = 8–12) and their caregivers participated. Most viewed asthma as a limitation to PA and could not identify a beneficial relationship between PA and asthma. Nonetheless, girls were enthusiastic about PA and shared strategies for managing asthma symptoms while exercising. Facilitators included outdoor access and social support, while barriers included program costs and safety concerns. Many girls said they would be more active with their mother/caregiver.

**Conclusions:**

Despite personal and structural barriers to PA, Black girls with asthma view PA as important for physical and social wellbeing. Mothers/female caregivers play a major role in motivating and creating opportunities for PA. Our findings inform efforts to promote PA in a vulnerable yet understudied population, including expanding asthma management education and leveraging mother‐daughter relationships to facilitate engagement in PA.

## 1 Introduction

For children with asthma, physical activity (PA) has been shown to improve symptoms and quality of life [1, 2, 3]. The World Health Organization (WHO) recommends at least 60 min of PA per day for all children aged 5–17 years, including for children with chronic health conditions like asthma [4, 5, 6]. However, evidence shows that children with asthma are significantly less active than their peers without asthma, often due to the belief that exercise restriction is an inherent part of having asthma [7]. Moreover, Black children face higher asthma prevalence and mortality, and are less likely to meet daily recommended PA compared to White children [8, 9, 10]. Black girls in particular are less physically active than their Black male and non‐Hispanic white peers, and their PA levels continue to decline through adolescence and beyond [11, 12]. Given the link between improved childhood asthma outcomes and PA, promoting such activity among Black girls with asthma is imperative.

PA interventions have been studied in people with asthma; however, they have largely focused on adults [13, 14]. Our previous work included the development of a 48‐week, walking‐based PA program for adult Black women with asthma [15]. Pilot study data indicated that the intervention was well‐received and led to significant improvements in asthma control [16]. Fewer interventions have focused on children, however. One study demonstrated that, after completing a 10‐week aerobic fitness program, 8–12 year old children with asthma showed improvements in lung function and quality of life [17]. Another study of children and adolescents with asthma found that, by the end of a 6–8 week aerobic and strength training program, participants had significant improvements in forced expiratory volume in 1 s (FEV1) [18]. While these approaches are promising, no PA interventions published to date have specifically targeted urban Black girls with asthma, a population that is at risk for lower PA and worse asthma outcomes.

Further, few studies have considered how mother‐daughter relationships can be leveraged as part of PA interventions for children with asthma [19]. For Black girls, mothers are thought to be the primary influence on PA by creating opportunities and modeling behavior [20]. Mother‐daughter PA interventions have been shown to increase PA levels among Black and Hispanic girls without asthma; however, such interventions have not been studied in girls with asthma [21, 22]. Given racial and gender disparities in childhood asthma outcomes and PA levels, it is important to explore how mother‐daughter relationships can support PA among Black girls with asthma.

To address the paucity of research on asthma and PA in Black girls, research is needed to understand beliefs and behaviors related to PA among Black girls, along with their female caregivers’ perspectives, including asthma‐specific PA knowledge, perceived risks and benefits of PA, environmental influences on PA, and role of mothers in promoting PA. This qualitative work provides insight into the lived experiences of urban Black girls with asthma and their mothers, informing the development of future PA interventions for Black girls with asthma that engage mothers as key supporters.

## 2 Methods

This qualitative study included semi‐structured interviews with girls treated for asthma and their mother/maternal figure at an urban academic medical center on Chicago's South Side, a community that is largely Black and impoverished. All interviews were conducted during fall 2024 by the research team. The study was approved by The University of Chicago Institutional Review Board (IRB23‐2062).

### 2.1 Population

Study participants were Black girls with asthma and their mother/maternal figure. The maternal figure was defined as biologic or adopted mother, aunt, grandmother, older sibling, or another female who is the child's primary caregiver. We use caregiver to refer to the maternal figure, biological mother or otherwise, throughout this paper. We focused on children 8–12 years old based on literature that shows mothers are the primary influence on Black children in this age range [20]. Specific inclusion criteria were: 1) child 8–12 years old at enrollment; 2) child and adult self‐identify as Black or African‐American and female; 3) healthcare provider diagnosis of asthma (as reported by caregiver); 4) adult self‐identifies as mother and/or female caregiver of child. Individuals were excluded if either mother or daughter did not read, speak, or understand English.

### 2.2 Recruitment

Individuals were recruited using a multi‐pronged approach based on our prior studies with this population. This approach involved flyers posted in clinical settings and direct outreach to patients identified from the electronic health record. Potential participants were sent a study invitation with a link to an eligibility survey via email, text, and/or mail. For those who completed the survey, the research team reached out to schedule eligible dyads for semi‐structured interviews. Prior to each interview, a research team member explained the study and obtained verbal caregiver consent and child assent. Additional participants were included until saturation was reached, or the point at which redundancy was noted in interviews.

### 2.3 Data Collection

Trained study staff conducted interviews using a semi‐structured interview guide (Appendix 1). The interview guide was created by study investigators using the Behavior Change Wheel (BCW) framework and pre‐tested for clarity and comprehension [23, 24]. The BCW incorporates two models of behavior change: COM‐B model, which suggests that change involves Capability, Opportunity, and Motivation, and Theoretical Domains Framework (TDF), which further categorizes cognitive, social, and environmental influences on behavior. Based on the BCW, interview topics included: knowledge of PA recommendations, perceived benefits and consequences of PA, asthma management skills during PA, environmental and social factors affecting PA, and maternal influences on PA. Interviews took place in‐person or remotely via a HIPAA‐compliant platform (Zoom), lasted 30 to 60 min, and were recorded. Each girl‐caregiver dyad was interviewed together. Subsequently, interviews were transcribed verbatim using Otter.ai software. Girls and caregivers each received a $50 gift card for their participation.

### 2.4 Data Analysis

To ensure interviews were adaptable to variability in participants’ responses, data analysis began after the first five interviews and continued after the interview phase was concluded. Data redundancy was apparent by the 20th dyad, indicating theoretical saturation had been met. Each participant was assigned a letter (D: Daughter or M: Mother) and a study identifier (ID; e.g. D1, M1). De‐identified transcripts were then coded for themes. The study team outlined a deductive coding scheme grounded in theory (based on COM‐B categories and TDF domains addressed in the interview guide). Using this scheme, two study staff members independently coded each transcript. The study team then reviewed transcripts together to discuss how codes were applied until consensus was reached. Analysis was completed using Dedoose (Los Angeles, CA).

## 3 Results

Participants included 20 dyads (Table 1), with 20 girls (age: mean = 9.9 years, SD = 1.33, range = 8–12) and 20 female caregivers (19 biological mothers, one grandmother; 14 were <44 years, 6 were **≥**45 years). The qualitative data were grouped into five broad themes: 1) PA knowledge and habits; 2) asthma triggers and management; 3) perceived benefits of PA; 4) barriers and facilitators to PA; and 5) PA and the mother‐daughter relationship. Table 2 summarizes key takeaways and quotes for each category.

**Table 1:** Characteristics of child and caregiver participants, *n* = 20 dyads.

Characteristics	Number	%
**Child's age (Years)**		
8	3	15%
9	5	25%
10	7	35%
11	1	5%
12	4	20%
**Time since asthma diagnosis (Years)**		
≤5	8	40%
>5	12	60%
**Caregiver's age (Years)**		
25–44	14	70%
45–64	6	30%
**Caregiver relationship to girl**		
Mother	19	95%
Grandmother	1	5%
**Caregiver's education level**		
High school Diploma/GED	4	20%
Some College/Associate's Degree	8	40%
Bachelor's Degree or Higher	8	40%
**Household size (Persons)**		
2–4	13	65%
5+	7	35%
**Yearly household income**		
<$25,000	6	30%
$25,000–$63,000	9	45%
>$63,000	5	25%

Characteristics	Mean	Standard deviation
**Child's report of asthma bother in past year** *		
(Scale 0–10)	5.11	3.20
**Child's report of difficulty walking at fast pace** *		
(Scale 0–10)	3.72	4.00

* *n* = 18 as two participants did not respond.

**Table 2:** Themes with key points and representative quotes related to physical activity (PA) and asthma among elementary school‐aged girls and their caregivers, *n* = 20 dyads.

Themes	Key points	Example quotes
Physical activity knowledge and habits	Girls described engaging in many forms of PA Most girls thought children with asthma should engage in the same forms of PA as unaffected children	“Everybody should be able to do some physical activity. I feel like everybody should be able to experience that.” (D3) “I would say, just because you got asthma, don't let it, like, make you stay down and not work out.” (D9)
Asthma triggers and management	Allergens, seasonal changes, and exercise were commonly reported asthma triggers Girls had multiple strategies for managing asthma symptoms, but usually did not use their inhaler before PA	“When the seasons change, her allergies flare up. Her allergies flare up her asthma.” (M41) “I get my asthma pump, take it and literally, just go back what I was doing. But if my asthma pump is not there, I literally sit down, breathe for a little bit…and then I go back where I was all started at.” (D21)
Perceived benefits of physical activity	Girls named many benefits of PA to themselves, families, and communities Girls and caregivers did not identify a link between PA and improved asthma symptoms	“Yes, it [PA] make me feel strong and happy.” (D22) “Her not being physically active, it doesn't affect it [asthma] that I see.” (M41)
Barriers and facilitators to physical activity	Barriers included lack of outdoor space, cost of exercise programs, safety concerns, and lack of an exercise partner or group Facilitators included school and social support and presence of neighborhood parks and free opportunities for PA	“They got the YMCA…you could get membership to get your physical activity. So I know that they got resources out here.” (M18) “We don't ride our bikes like we used to, because there's been shootings in the area, so we kind of stick a little closer to home.” (M17)
Physical activity and the caregiver‐child relationship	Girls and caregivers described multiple ways they motivate each other to engage in PA Girls said they would be more active with their mother or caregiver	“I'll grab her hand and then I'll ask her to run and then she'll say that she can't run, but sometimes she said, she can run.” (D13) “She [caregiver] tell me that I'm good at doing what I like to do, and it inspires me more.” (D6)

Abbreviations: D, Daughter; M, Mother.

### 3.1 Physical Activity Knowledge and Habits

Girls described regularly engaging in many forms of PA, including running, walking, riding bikes, cheerleading, dancing, and playing sports. Some girls described incorporating PA into their routines at home, such as climbing stairs while doing chores or playing with siblings and pets. In addition, participants described social media playing a role in daily PA. “She dances…every day on TikTok,” said one caregiver. “I mean, it's not long, dancing, but it's at least she's physically moving for a few minutes.” (M3).

When asked about the recommended amount of daily PA for children, girls provided estimates ranging from a few minutes to several hours per day. Six girls said less than an hour per day was recommended, eight said one to 2 h, and two said more than 2 h. Two girls stated recommendations differed depending on asthma status, with both estimating that children with asthma should engage in half as much daily PA as children without asthma.

Many girls felt that children with asthma should be able to engage in the same forms of PA as children without asthma. “They can do the same thing other people without asthma do,” said one girl. “Just bring your inhaler.” (D12). However, some thought that children with asthma should engage in lighter, “not too intense” forms of PA, such as stretching, walking, or low‐intensity sports (D40). “Just taking walks,” suggested one girl (D19), while another said, “Maybe simple things, like riding a bike and just walking.” (D15). One caregiver provided a more balanced view: “I think that they could do just about everything within moderation, be able to monitor their symptoms.” (M41).

### 3.2 Asthma Triggers and Management

When girls were asked how much their asthma bothered them over the past year (0–10; 0 = *not bothersome at all*, 10 = *bothersome all the time*), the mean score was 5.11 (SD = 3.20). Many girls and caregivers pointed to seasonal allergens and weather changes as asthma triggers. Some described concerns about cooler weather, with one caregiver reporting that “in the fall, winter is kind of when it picks up and we have to be very cognizant of her breathing and illnesses.” (M17). Caregivers also said that allergens, like pollen and pet dander, play a role in provoking asthma flares. “She's allergic to dust, ragweed, elm trees, which she's around constantly,” said one caregiver (M3).

Most girls also pointed to PA as a trigger for asthma symptoms. They described experiencing shortness of breath, wheezing, and chest tightness, sometimes so severe they had to stop exercising altogether. One caregiver said, “It's treading lightly with the activity and how much she can do and for how long…she wants to do a lot, and sometimes that holds her back.” (M25). However, many girls expressed determination to engage in PA despite the perceived risk of asthma exacerbation. “I know three people with asthma,” said one girl (D3). “We don't let asthma stop us…we're both very physically active.” Another girl was motivated to engage in PA so she could serve as a role model for others with asthma: “I can show them how I went through asthma and how I was able to manage it.” (D8). When asked to rate the perceived difficulty of walking at a fast pace (0–10; 0 = *not difficult*, 10 = *very difficult*), girls reported a mean score of 3.72 (SD = 4.00).

To maintain participation in PA, girls possessed multiple strategies, both medical and non‐medical, for managing asthma. Medical strategies included using their rescue inhaler or other medications such as prednisone or diphenhydramine. Non‐medical strategies included taking a break, catching their breath, and drinking water. Of note, most girls did not report using their inhaler before engaging in PA. “I've never really learned to use my inhaler before stuff,” said one girl. “Like, people have just told me, if I need it, use it.” (D3). Some caregivers encouraged girls to take more control in caring for their asthma. “I think the older she's getting, she's learning how to manage the symptoms a little bit more on her own,” said one caregiver (M3).

### 3.3 Perceived Benefits of Physical Activity

Girls listed numerous benefits of PA, not only to themselves but to their families and communities. Personal benefits included having fun, competing, and staying healthy. Family benefits included bonding through shared exercise and playing sports or performing for their family to watch. “They always cheer me on,” said one girl. “And after my competition…sometimes they give me flowers. So I think they like watching me perform.” (D3). Community benefits included participating in events organized around PA and helping to promote physical wellness. One caregiver described the importance of combating cultural stigma around exercise, saying, “I think the benefit of having these social activities around physical fitness, and mainly in the African‐American community, because that's not something that we were raised on. And for us, physical challenges almost seem like something negative.” (M3).

While participants identified various benefits of PA, most girls were unable to identify a connection between physical inactivity and worsening asthma symptoms. One girl said, “If I'm not doing anything, like running or like physically, then I'll just be at home and, like, my asthma will be perfectly fine.” (D3). However, one caregiver observed negative effects of physical inactivity: “We do have a peak period between, like, January and March, where she is not physically active…I do kind of see a difference in her. She actually does get sicker too, I have to honestly say.” (M3).

### 3.4 Barriers and Facilitators to Physical Activity

Facilitators to PA included availability of outdoor spaces and community resources. “We definitely have nice sidewalks,” said one caregiver. “We have bike lanes. We're not too far from a park, which just recently put in a new walking path. And they also have, of course, it's a park district, so they have those classes.” (M17). Social support from friends, family, and school staff was also a factor that encouraged girls to exercise. “Her school is very supportive,” said one caregiver. “They are aware that she has asthma, so we shared the asthma action plan with them…we all work together as a team.” (M2). Another girl described support from her softball team: “They've learned that I had asthma, and so they sometimes let me walk. Like yesterday, they let me walk because I couldn't run.” (D3). Girls were particularly motivated by the fear of missing out on exercise‐based social opportunities. “She will monster through into the last step, if she can, just to be part of the group,” said one caregiver (M3).

Several caregivers identified environmental and social factors that made PA more difficult, however. These barriers included lack of outdoor recreational spaces, financial cost of exercise programs, and concerns about violence. “The park that was around here was attached to a school, so they shut it down,” said one caregiver (M21). “It was a community thing. Over the weekend, we would go over there and meet up with other parents and kids.” Some caregivers said they have to travel to areas of the city that are safer or have more green space to exercise. “We don't live in the best of neighborhoods, and so sometimes…we literally have to go across town,” said one caregiver (M2). Additionally, while some girls endorsed social support for PA, others felt that they needed a partner or consistent group to be more active. With a partner, said one girl, “you won't be by yourself and you'll be okay.” (D15). One caregiver said that a “facility where it was just children her age and they were all kind of engaged in the same activity” would encourage her daughter to exercise more (M3).

### 3.5 Physical Activity and the Mother–Daughter Relationship

Of the 20 girls interviewed, 18 said they currently exercise or would be willing to exercise with their caregiver. Dyads described participating in shared activities like going for walks, riding bikes and jumping rope. “She is so much fun to work out with,” said one caregiver about her daughter. “She makes it more of a game, instead of, like, actual workout.” (M40). Girls and caregivers reported that they suggest types of PA for the pair to engage in together. “I'll just say, do you want to kind of take a walk with me?” said one girl (D8). One caregiver said, “I tell [my daughter] to get up and move around more, get up and do something.” (M17).

Beyond suggesting activities, girls and caregivers described other ways that they motivate each other. Many girls said that their caregiver encourages them with positive feedback. “When we're at the volleyball games, I could hear her cheer for me,” said one girl. “When I also did basketball, she was, like, the last one there cheering for me.” (D40). Some caregivers also stated they found motivation in seeing their children participate in sports and exercise. “Just endurance, perseverance, confidence, you know…[my daughter] puts her whole everything into it, and I'm inspired by that,” said one caregiver (M2). Another caregiver felt it was important to instill a sense of confidence and belonging in her daughter, despite her asthma: “I don't want her to feel like she's not normal, like all the rest of the kids, because she has to stop doing the activity or slow down because…her chest might be tightening or she's out of breath,” she said. “I still want her to understand that it just takes time for her because she has asthma, versus her feeling like she can't do it at all.” (M6).

## 4 Discussion

Our findings contribute a novel perspective from urban Black girls to the literature on PA and asthma in children. Understanding this population's views on engaging in PA with asthma is an important step toward enhancing physical activity and addressing disparities in childhood asthma morbidity. We also found that the mother‐daughter dyad can be beneficial in promoting exercise among girls with asthma, laying the groundwork for future PA interventions in this population.

Overall, asthma was viewed as a restrictive factor for PA, with few participants able to identify a beneficial relationship between PA and asthma. This finding is consistent with previous qualitative research on attitudes toward PA among children with asthma and their caregivers. In a study of parents of children with asthma in the Bronx (New York), participants reported telling their children to take frequent breaks from PA or abstain altogether to avoid provoking asthma symptoms [25]. Two UK studies similarly found that some children and caregivers believed shortness of breath was a sign of asthma exacerbation, rather than a normal part of being active [19, 26]. Lack of knowledge has emerged in the literature and in our analysis as an obstacle to achieving recommended levels of PA, suggesting that education is needed on the benefits and safety of PA participation for children with asthma. Notably, although many caregivers in our study held the belief that PA worsens asthma, they largely encouraged girls to engage in PA and often took inspiration from watching them participate. This support is promising and suggests that a PA intervention for Black girls with asthma would be viewed positively by caregivers.

In addition to the perceived limitations of asthma, our study population faces structural inequities that limit access to becoming and staying active, including safety concerns, financial strain, and lack of outdoor space. Financial and safety issues have also been identified as barriers to PA in previous studies that included children with asthma in under‐resourced settings [25, 27]. In contrast to these challenges, most participants noted familial support to be a facilitator to PA. Girls felt encouraged by their caregivers and communities to demonstrate their physical abilities. This finding aligns with a prior study of child‐caregiver dyads – including Black, White, and Hispanic families – which found that family praise and being active with family members were both motivators for PA among children with co‐morbid asthma and obesity [28]. Similarly, most girls we interviewed said they would be active with their caregiver, yet exercising together was not a regular habit for many dyads. Our study confirms that previously documented associations between joint caregiver‐child PA and increased PA hold true among Black girls with asthma and their female caregivers. We conclude that Black girls with asthma and their female caregivers would benefit from structured opportunities to participate in PA together.

In our study, girls were enthusiastic about PA as a prominent part of their physical and emotional wellbeing. Prior studies show mixed results in terms of how children with asthma perceive their physical capabilities. One meta‐analysis concluded that children with asthma sometimes experience shame and isolation during sports due to a sense of being different than their peers [29]. In qualitative studies of children and adolescents with asthma, participants shared that exercise was still possible and cited personal enjoyment and socialization as reasons to engage in PA [26, 30]. Girls in our study also referenced these benefits of PA. However, their responses alluded to another motivator for PA that has not been described in the literature: the desire to demonstrate they were capable of exercise despite their asthma diagnosis. Black girls’ desire to persevere through asthma suggests that a PA intervention would be well‐received by this population, while also highlighting the importance of designing interventions that build on Black girls’ resilience and belief in themselves.

Limitations of this study include a small sample size, which represents perspectives that are specific to a population of Black elementary‐school aged girls and their caregivers in the urban setting of Chicago and thus may not be generalizable to all Black girls with asthma. Although participants were told there were no right or wrong answers, social desirability bias may have influenced how they reported PA behaviors. Nonetheless, our analysis builds on a growing base of knowledge, contributing the voices of a population who has not regularly been included in research.

Overall, this study shows that Black girls with asthma hold eager, positive attitudes toward regular PA participation and employ multiple strategies to manage their asthma during PA. These findings will inform the development of a new physical activity program for this population, incorporating asthma education and including mothers and female caregivers to promote sustainable engagement in PA. Such interventions may improve disparities in physical activity and asthma outcomes in historically marginalized populations.

## Author Contributions

Sharmilee M. Nyenhuis and Anna Volerman conceptualized, designed, and acquired funding for the study. Deborah Salvo and Shelby Langer assisted with interview guide development and data analysis. Sharmilee M. Nyenhuis, Anna Volerman, Aero Cavalier and Ellen Davis collected data. Nora Spadoni, Aero Cavalier, Ellen Davis, Sharmilee M. Nyenhuis, and Anna Volerman analyzed data. Nora Spadoni drafted the manuscript. All authors revised the manuscript and approved the final version.

## Ethics Statement

This study was approved by The University of Chicago Institutional Review Board, IRB23‐2062.

## Consent

Consent was obtained from parents or caregivers of child participants prior to entering the study. Assent was obtained from child participants.

## Conflicts of Interest

The authors declare no conflicts of interest.

## Supporting information

Supplementary Information.

## Data Availability

Research data are not shared to protect the privacy and confidentiality of participants.
