# Supplementary material for: Beliefs and Behaviors Related to Physical Activity in Black Girls With Asthma
Source: Pediatr Pulmonol. 2026 Feb 5;61(2):e71497. doi: 10.1002/ppul.71497 (PMC12875540; doi:10.1002/ppul.71497)
Supplement: Supplementary file 1 — Supplementary Information. [file PPUL-61-0-s001.docx]

| **Appendix 1. Theoretical domains included in interview guide with questions for girl-caregiver dyads** | | | |
| --- | --- | --- | --- |
| ***Capability*** | ***Opportunity*** | ***Motivation*** | ***Corresponding Questions*** |
| Knowledge |  |  | On a scale of 0-10, how much has your/your child’s asthma bothered you in the past year?  What things set off your child’s asthma or make their asthma worse? What things improve your child’s asthma?  How much physical activity do you think is recommended for children per day?  How much physical activity do you think is recommended for adults per day/week?  What types of physical activity do you think are recommended for people with asthma? |
| Nature of the behaviors and behavioral regulation   - Action planning - Self-monitoring - Self-confidence - Perceived competence |  |  | What is your favorite type of physical activity?  What steps would you need to take to prepare to start doing physical activity on a regular basis?  What do you currently do if you have asthma symptoms while being active?  What are your biggest challenges for engaging in physical activity regularly?  How difficult is it for you to walk at a fast pace on a scale of 0-10 with 0 being not at all difficult to 10 being extremely difficult? Why? |
| Attention | Environmental context/resources |  | Have you noticed any efforts in your community to promote/ facilitate/encourage physical activity?  To what extent does crime or violence in your community prevent you from engaging in physical activity?  To what extend does high traffic/lack of paths or sidewalks or parks in your neighborhood prevent you from engaging in physical activity? |
|  | Social influences | Motivations and goals | What percentage of women/girls with asthma do you think engage in physical activity regularly?  Do you feel like you are more physically active when you do it with others (friends, family members) OR when you are alone? Or the same?  Would you do physical activity with your daughter/mother? If no, why?  How would your daughter/mother motivate you to engage in physical activity?  During the past three months, my family (or members of my household) or friends:   - Exercised with me. (True/False) - Gave me helpful reminders to exercise. (True/False)   Please tell me the degree to which you agree, neither agree nor disagree, or disagree with the following:   - My family is supportive of me exercising. - My friends are supportive of me exercising. |
|  |  | Beliefs about capabilities/  consequences | How do you think not being physically active affects your child’s asthma?  What problems have you encountered in the past when doing physical activity?  What do you think are the benefits of physical activity:   - To you personally? - To your family? - To the wider community? |
